# Elements of Operative Midwifery, &c.

**Published:** 1826-01

**Authors:** 


### *Elements of Operative Midwifery, &c. &c. &c.* By D. D. DAVIS, M.D. &c.

[Continued from last Number of this Journal.]

THE part that we have now to examine of Dr. Davis's work relates chiefly to the more difficult and least frequent operations of midwifery; namely, those in which, on accouut of a disproportion between the capacity of the maternal pelvis, and the bulk of the child to be propelled through it, or, on account of the malposition of the child, the use of instruments becomes indispensable for the preservation of the mother's life, while, in many cases, there is no probability, and, in some, no possibility of saving that of the child, without exposing the mother to the utmost danger. The fact that such serious and difficult cases are of comparatively rare occurrence, renders it impossible for the generality of medical practitioners to acquire proficiency in the treatment of them from actual practice, and, therefore, it becomes an imperative moral duty for every gentleman who intends to practise midwifery, to embrace with earnestness every other practicable means that may present itself of acquiring a competent knowledge of this department of his profession, if he would wish to be able to recognize such cases when they do occur, or to treat them in a manner creditable to his reputation, consistent with his peace of mind, or honourable to the art. Every surgeon, and, indeed, every physician ought to possess a knowledge of this branch of midwifery, as it is chiefly in cases of such extreme danger, where delivery is impracticable without operative assistance, that men presumed to possess superior knowledge are resorted to for assistance, and every professional man may chance to be placed in circumstances where it could only reflect discredit upon him, if he were either unwilling or incompetent to afford such assistance.

The third section of the work under review, is entitled—"Remarks on the Use and Special Properties of the Long Forceps; with Suggestions for certain other modifications of obstetric instruments." The long forceps, as the term is understood in this country, is nothing more than a pair of forceps of sufficient length to admit of being applied to the fœtal head before it has entered the brim or superior aperture of the pelvis. It is generally admitted that our justly celebrated countryman, Dr. Smellie, was the first to propose the application of forceps to a head in this situation. It may be assumed as generally true, that, when there is no great disproportion between the size of the fœtal head and the capacity of the maternal pelvis, the force of the parturient powers will be sufficient to overcome the resistance opposed to the passage of the head. Indeed, if we consider the prodigious and almost incredible power which nature is capable of exerting in such circumstances, equal, as it has been known in some instances, to drive the child's head through the wall of the vagina and integuments of the perineum, when prevented, by preternatural rigidity, from dilating or passing through the os externum; it is difficult to suppose a case in which, such powers having failed to force the head past the brim of the pelvis, it could be safe to attempt to bring it down, in an entire state, by the aid of any instrument whatever. It may, therefore, be considered as a general rule, that, in cases where confinement of the brim is so considerable as effectively to resist the efforts of the natural parturient powers to force the head into the cavity of the pelvis, it will not be possible to accomplish that object by artificial means, compatibly with the preservation of the child, and without extreme danger to the mother. This general rule, however, is not without exceptions, and these exceptions are treated of in a clear and highly satisfactory manner by Dr. Davis in the section now under review.

There are four classes of cases in which, according to our author's opinion, the long forceps might occasionally, under particular circumstances, be advantageously had recourse to:— 1st. Limited confinement of the brim of the pelvis. 2dly. Certain cases of uterine hæmorrhage. 3dly. Rupture of the uterus; and—4thly. Syncope occurring during labour.

It is to be presumed that the very slow and gradual procedure by which, in cases of slight confinement of the pelvis, nature effects the change in the form of the fœtal head, which is indispensable to its transmission through the pelvis, is more favourable to the welfare of the child than any equal degree of compression that might be more speedily produced by other means. Dr. Davis is even doubtful whether a degree of compression adequate to produce the requisite diminution of bulk; that is, a diminution equal to that which is often effected with impunity by the unassisted agency of the natural powers, could be applied, with safety to the child's life, by any artificial means whatever; and, therefore, he says—

"It should be considered as generally improper to have recourse to the use of the long forceps, as long as nature can be entrusted to exert her efforts without compromising the safety of the mother; and while she holds out a fair promise, that eventually, and compatibly with a living birth, she may triumph over her difficulty. Under good general management of unfortunate labours of this description, the natural struggles may often be supported with perfect impunity to the soundness and safety of the organs concerned for very many hours, and until the uterine efforts shall eventually become so feeble and ineffective, as to indicate the expediency of operative interference, as a measure which, at any rate, it would be no advantage longer to postpone. Again, we may easily conceive of cases where the natural powers, under such a direction as we have supposed, might all but suffice to effect the propulsion of the fœtal head into the pelvis, and yet fail, or become feeble, at a most critical juncture of a labour, when very little additional effort might have overcome the whole difficulty.

"Moreover, in some other cases of limited confinement at the brim of the pelvis, we occasionally encounter an apparent deficiency of uterine action, as if nature was really conscious of her difficulty and danger, when she might probably succeed in the ultimate propulsion of the fœtal head into the pelvis, were her powers to be fully called forth and exerted."

From the very limited range of its possible utility in cases of confinement of pelvis, Dr. Davis justly observes, that the long forceps is an obstetric power that seldom ought to be had recourse to, even in severe labours; and one rather to be sometimes cautiously tried by practitioners experienced in the more common duties of instrumental midwifery, as a preliminary measure and possible preventive of the more dreadful operation, craniotomy, than to be ranked among the ordinary resources of the art.

The second class of cases in which the long forceps may possibly be usefully employed by a dexterous operator, are those complicated with uterine hæmorrhage, where the orifice of the uterus is amply dilated, the pelvis of good dimensions, and the child's head presenting at the brim. In such cases, the use of the long forceps might at least deserve to be weighed in comparison with the operation of turning.

With respect to rupture of the uterus as an indication for the use of the long forceps, that dreadful misfortune usually occurs either before, or just after the entrance of the head into the brim of the pelvis, and before it has descended so low as to be within reach of the short forceps: and though the long forceps ought not to be entirely overlooked on such occasions, it would generally be extremely difficult to apply it with advantage, on account of the recession and unsteadiness of the head, after the partial or complete escape of the child's body from the cavity of the uterus into the general cavity of the abdomen. Moreover, defective capacity of the pelvis at the brim, being the most frequent cause of the accident in question, must, of course, be unfavourable to the successful use of the long forceps in the majority of cases of rupture of the uterus.

English writers on midwifery have mostly rejected the long forceps altogether, and have, therefore, generally given no directions as to the mode of applying it, whilst the rules prescribed for its use by the majority of foreign authors, are such as it would frequently be extremely dangerous, and, in some cases, totally impracticable to observe. This observation of our author is more especially applicable to the direction given for applying the blades of the instrument in all cases over the ears and sides of the head, for Dr. Davis is of opinion that the majority of cases, usually deemed objects of forceps' operations, before the descent of the child's head into the cavity of the pelvis, are such as will not admit of the blades being applied in that manner; that is to say, are cases in which the head presents, with the forehead towards one side of the pelvis and with a deficiency of space between the symphysis pubis and promontory of the sacrum.

When the head presents at the brim of the pelvis with the forehead towards the sacrum, or towards the symphysis pubis, the common long forceps may be applied, when circumstances exist to render a resort to this instrument expedient, much in the same manner as the short forceps are applied, viz. with one blade at each side of the pelvis, and adapted to each side of the head. In some such cases Dr. Davis thinks that it might facilitate the object in view, if we had a pair of forceps with one blade broad and the other narrow.

Supposing the instrument introduced in a case of the first position, that is, with the forehead towards the sacrum, our author gives the following cautious directions for its use.

"After locking the instrument with great caution, so as to avoid the possibility of including any important structure within its grasp, we draw down with cautious firmness, in strict correspondence with the axis of the pelvis. A few repetitions of efforts of this kind, co-operating with the simultaneous exertions of the natural powers, will soon enable us to form some judgement of the probable issue of our interference. An obstinate immoveableness of the head, no perceptible descent or increase of engagement of it within the cavity of the pelvis, being effected by such cautious endeavours, would naturally indicate the duty, at least, of pausing; and very probably, that of desisting altogether from further interference. To make an effort of this kind, with a view to the preservation of the child's life, with extreme caution, and yet with as much firmness as may be compatible with the perfect safety of the mother, is all that can be done to meet the utmost demands either of humanity or professional duty. To give every possible chance for the success of this movement, it might be sometimes useful to attempt to incline the child's face towards one of the sacro-iliac junctions, which might probably ensure for it some little more space than it could have when directly opposed to the promontory of the sacrum. This attempt failing as to one side, it might be right to repeat it as to the other. Having brought down the forehead below the level of the posterior part of the linea ileo-pectinea, it will then become necessary to carry the face to the hollow of the sacrum.

"After having thus, with the greatest imaginable caution, completed the adduction of the fœtal head into the cavity of the pelvis, it will be proper, in a great majority of cases, to withdraw the instrument forthwith and altogether, and to surrender the remaining part of the labour to the disposal of nature."

The reasons for, and exceptions to this last rule are fully detailed by our author, as part of his instructions for the use of the common forceps.

Cases of the second position, namely, where the forehead is towards the symphysis pubis, are, of course, more difficult to manage, and less likely to be benefitted by the long forceps, than those of the first. Dr. Davis says decidedly that it is idle to pretend, as some authors have done, that such cases are convertible into first-position cases, by turning the forehead from the pubes round towards the sacrum, with the fingers alone; and that, if it were practicable to effect this rotation with the forceps, it could not be done without giving a fatal twist to the child's neck.—We must, therefore, in his opinion, meet the case as it really is, difficult to manage, and uncertain as to its issue, and endeavour by strict attention to the directions and cautions which he clearly lays down, to conduct it to a safe termination, without attempting to turn the face towards the sacrum.

"In nine out of ten cases of arrest, or difficult entry of the child's head at the superior aperture of the pelvis, the actual position of it, is that of the forehead and face to one side, and of the occipito-vertical part of it to the other. With a trifling inclination of the back part of the head towards the front of the pelvis, this is also almost always the relative situation of the fœtal head during the earlier stages of natural labour. We have, indeed, seen that, in ordinary cases, the head, during its natural descent into the pelvis, undergoes a gradual and spontaneous change of position; so that its vertex, which at first presents in more or less perfect correspondence with one of the acetabula, is gradually and imperceptibly veered by a gentle rotatory movement towards the front, so as to be made to engage, with great advantage to its progress, under the arch of the pubes. It is evident, however, that in cases of defective capacity of the pelvis, and especially in those of labours rendered difficult by defect of space in the direction of the conjugate diameter, this gradual change of position cannot take place; and, therefore, that the fœtal head must remain situated transversely across the superior aperture, either until the difficulty shall have been surmounted by a strenuous and long-continued exertion of the natural powers, or until art shall have interfered to advance its progress. Now, if the arrest of the head thus situated at the superior aperture of the pelvis be really the effect of defective space in the direction of the conjugate diameter, a very little consideration will make it apparent that the long forceps of the ordinary construction is not an instrument really calculated to meet the demands, by obviating the difficulty of such a case. In the application of such a pair of forceps either the blades must embrace the lateral parts of the head, or those of the forehead and occiput. Of the respective claims to our preference of each of these modes, I am aware that there are extant some plausible descriptions: but let these pretensions be made the subject of a little examination. If the pelvis be already too small to admit the child's head to engage in it in the position which it is presumed to occupy, which is that of a more or less perfect correspondence of the transverse diameter of the child's head to the conjugate diameter of the pelvis, how and where is it possible to find room for the subsequent occupancy of the two blades of a pair of forceps? Those blades in the instance of the most modern and most approved French forceps are, each of them, a full sixth of an inch in thickness; and let the individual instrument preferred be ever so lightly constructed, it must occupy a certain amount of space; whereas the very condition of the case under consideration is an actual deficiency of space, *i. e.* the absence of a sufficient amount of space in the direction of the short diameter of the pelvis, even for the engagement of the head alone; without any addition to its bulk by the application to it of two blades of a powerful steel instrument. May I not also very properly insist upon the great and manifest danger of forcibly using a bulky and powerful metallic instrument in a situation so important, as to the nature of the structures concerned, and so confined as scarcely to admit of a remote chance of their escaping injury?

"These facts being, in my opinion, indisputable, I am much disposed to the belief, that the long forceps has really never been properly used in the manner and circumstances here supposed; and that it never has enabled the practitioner to deliver his patient on the principles assumed, excepting in cases where nature, unassisted, would in all probability have performed the duty much better, and excepting also in some rare cases of complicated labours, unaccompanied by any deficiency of space. The mere possibility of introducing the long forceps in the manner and circumstances here described, viz. that of passing up its two counterparts, one after the other, along the opposite sides of a head too large to engage in the cavity of the pelvis, is at obvious variance with the asserted conditions of the case; whilst, on the other hand, the adduction of the fœtal head into the pelvis, subsequently to the reduction of its bulk by the compressing action of the forceps, must at any rate be an extremely tedious process, and, therefore, equally at variance with the vaunted facility, rapidity, and prosperous issues of some brilliant operations of this kind, of which we have too many published descriptions.

At all events, I consider the old long forceps as decidedly unsuitable for the relief of cases of arrest of the fœtal head at the superior aperture of the pelvis, under the circumstances of either of its transverse positions. Accordingly, I have to submit to the approbation and cautious trials of the profession, a form or modification of a pair of forceps, which may be used under these circumstances with considerable effect; and certainly, if dexterously used, without any risk of doing an injury to the mother."

The author here refers to the plates for several views of this new variety of obstetric power. One of the plates exhibits a beautiful representation of the instrument in application to a fœtal head supposed to be arrested at the brim, with the forehead towards the left side of the pelvis. It consists of two branches, one of which is much longer than the other, and is covered with leather, lined on its concave surface with a padding of the softest flannel, which is intended to protect the face, over which this blade must be applied. About an inch and three quarters from the extremity of this blade there is a joint or hinge, which admits of the curve of the blade being increased to a certain extent, so as to bring that part which is beyond the hinge into close apposition with the mouth and chin of the child. The flexion or extension of the moveable part of the blade is made at the will of the practitioner, by means of a small button which is situated on the shank of the instrument. It is difficult to convey a very distinct idea of the mechanism and application of this instrument without referring to the plates, but let the reader suppose the occipital vertex of the child's head to present at the brim of the pelvis with the forehead towards the left side, and the blade of the forceps just described, to be introduced along the pelvis and crown of the child's head until that part of the blade where the hinge is situated has passed the bulge of the forehead, and that the moveable part is then bent to the greatest extent and brought into close apposition with the lower part of the child's face, and fixed in that position by a notch near the button on the shank of the blade.—When this is done the instrument becomes a hook or adductor of very considerable power, and when the short blade is introduced in application to the occipital region of the head, it serves as a fulcrum and antagonist to the long blade, preventing the head from slipping from its grasp and from being pressed against the right side of the pelvis; but, with this instrument there is no counter-pressure applied to opposite parts of the head as with common forceps, for the novelty and merit of the instrument consists in its attracting power being applied to a surface nearly opposite to the presenting part of the head, and in its not occupying any of the space, already supposed to be deficient, between the promontory of the sacrum and symphysis pubis. When the head has been once drawn past the brim of the pelvis the operator may gently endeavour to turn the face into the hollow of the sacrum, or he may remove the long forceps and leave this turn and the rest of the labour to be effected by the natural powers, or, if necessary, he may introduce the short forceps with blades of unequal length, already described as having been invented by Dr. Davis for the special purpose of turning the face from either side of the pelvis into the hollow of the sacrum.

With respect to face-presentations, Dr. Davis is of opinion, that in a great majority of them, the natural powers are sufficient to effect the delivery compatibly with the safety both of the lives and structures interested in the struggle, and, at all events, compatibly with the preservation of the mother's life. This, he thinks, must at least hold true in three out of the four positions into which face-presentations are usually distributed by authors, viz. those with the chin directed to the front or to either side of the pelvis. When a practitioner is called to a case at an early period of the labour, and ascertains that the face presents, before the discharge of the liquor amnii, or soon after the rupture of the membranes, our author very properly recommends the immediate performance of the operation of turning, as the best measure that can be adopted both for mother and child. In cases where the head has descended low down into the pelvis, where the uterus has been long firmly contracted upon the body of the child, and where the chin is directed to one side of the pelvis, he thinks that by cautious management it might be turned by means of the fingers, so as to come out under the symphysis pubis, or, if this failed, the same object might perhaps be effected by the aid of forceps with blades of unequal length. When the chin is brought to correspond with the arch of the pubis, the common short forceps might, if necessary, be advantageously employed to assist in completing the extraction of the head.

When the face presents with the chin towards the sacrum, it has heretofore generally defied the art of midwifery to save the life of the child, although Dr. Davis quotes a case of Mr. Perfect's, in which, according to that author's account, he effected the safe delivery by means of common forceps. Dr. Davis thinks that, in some cases of this presentation, it might not be impracticable to effect such an entire change in the position of the head as to bring the vertex to present instead of the face. For this purpose he suggests the trial of a common vectis, furnished with six or seven short, round, and very small teeth, projecting obliquely from the internal surface of the clams of the blade at an angle of about 35 degrees, looking towards the handle of the instrument. The length of the teeth not to exceed one-tenth of an inch, and their thickness to be equal to that of a small pin.

"Might not an instrument of this kind, carried up into the pelvis, in any direction in which it could most conveniently pass, applied to the occiput of the child, and there made to transfix the integuments of the part, enable an adroit operator to effect the whole change of the position of the fœtal head that might be required to place it within the influence of the natural agents of the parturient function? or, at least, so far to improve its position as to make it perfectly accessible to the purchase of the common forceps? To this procedure it might justly be objected, that it supposes the infliction in the living child of several painful and dangerous wounds; which might prove fatal, either speedily from the loss of blood, or, at a later period, from the accession of violent inflammation and gangrene of the wounded part. I have, indeed, no evidence of facts to offer in opposition to the force of these objections. With respect to the painfulness of such an operation to the child, I think, (notwithstanding the extraordinary opinion of Dr. Osborn, that children not really born, though indisputably alive, cannot feel) that the fact must be fully admitted.

"As to the danger from loss of blood, from five or six simple punctures made into the occipital scalp, with such round and small steel points as we have supposed, it seems to me that it should be estimated at very little. The instrument would of course be fixed at a remote distance from the presenting part of the head, and, therefore, into a structure at least not more than naturally charged with blood. With respect to the accession of gangrene after the operation; that would greatly depend upon the general state of the child at its birth as to constitutional strength; and upon its being properly nursed, and in all respects well managed or otherwise, immediately, and for the few first days after its birth. If it should escape with its life, the preservation of that life, in a certain proportion, at least, of the class of cases under consideration, might be placed as clear gain to the account of the operation."

Ear and side of face presentations are of very rare occurrence. Dr. Davis has never met with one example in his private practice, and, in that of the Royal Maternity Charity, only one in nine years.—The almost exclusive indication of treatment in such cases, is that of bringing down by a gentle rotatory movement of the head on its occipito-frontal axis, its vertex, or any other part in the immediate neighbourhood of the vertex, towards the bottom of the pelvis. This may be attempted in the first instance by the fingers, or if these should fail, then by the common vectis applied to that part of the head immediately above the superior ear.—For more ample and particular instructions relative to these cases we must refer to Dr. Davis's work.

In the course of some general practical observations on the use of the forceps, the whole of which are well entitled to the careful and repeated perusal of practical accoucheurs, our author remarks that the accession of an obstinate diarrhœa soon after the

"Consummation of severe labours, whether instrumental or otherwise, may, with great reason be apprehended to arise from an inflamed state of the mucous membrane of the rectum, and to indicate a dangerous degree of contusion of the recto-vaginal septum."

He quotes cases from other authors in illustration of this remark, and gives the following one from his own practice.

"The lady had been married thirteen years, and this was her first labour. The parts were rigid, and the pelvis, without being distorted, was not ample. The head had been bearing strongly, and for many hours, on the parts lining the pelvis, before I determined to give artificial assistance; and the head was eventually extracted, not without the exertion of more force than I should often choose to apply, though incomparably less than represented to have been used in some of the above cases.* On the third day after the delivery, the patient, already the subject of much febrile action, was attacked with a severe rigor. She was harrassed for many days afterwards with a most troublesome and exhausting diarrhœa. On the eighth day after her delivery she sustained an entire abolition of her mental faculties;

---

* The author here alludes to some cases quoted from Perfect and Smellie, which we have not extracted.—*R.*

which, however, in about fifty hours afterwards, were again suddenly restored to her, as an effect apparently of a long sleep procured by a dose of sixty drops of Battley's liquor opii sedativus. After having been vibrating in a very nice balance, I think I may say for an entire month, she eventually recovered; not a little, as the reader may well suppose, to my satisfaction."

We have extracted this case as shewing the occurrence of diarrhœa after a severe labour, in conjunction with other formidable symptoms, and we would take this opportunity of recommending, particularly to the attention of the experienced reader, the practice resorted to so successfully in this case by Dr. Davis, of giving a dose of opium large enough to suspend, in profound sleep, and, it may even be said, effectually to extinguish an incipient attack of mental derangement. The practice of administering large doses of opium in mania is not general, nor, as we apprehend, is its value duly appreciated. It is one, however, which, with proper discrimination, promises much good.* To the account of the above case Dr. Davis subjoins the remark that in consultation practice, he has also seen a good many cases of the accession of diarrhœa after severe labours, and especially after the use of the forceps; and, upon the whole, he considers diarrhœa, when it occurs in such circumstances, as a symptom indicative of the greatest danger.

The following truly practical observations on the general treatment necessary to be observed after forceps' operations cannot be perused with more attention than they deserve.

"Operations with the forceps are seldom performed so early in a labour as to ensure a perfect freedom from the risk of subsequent reaction; even if we were to suppose that instruments of this class could always be used without materially increasing that risk. Hence, the treatment of puerperals, under these circumstances, is usually a duty of much more than ordinary magnitude and responsibility. If the labour shall have been one of considerable severity and duration before the decision is taken to have recourse to artificial assistance, and then the delivery is effected not without some difficulty, we might generally calculate on the subsequent accession of a smart symptomatic fever. Now it cannot be doubted that a fever of this description would have for its principal and proximate cause a state of great tenderness, approaching to that of contusion, and a highly inflammatory condition of the structures within the pelvis and the lower parts of the abdomen. But such a condition of the structures in question could not be allowed to continue long, and, perchance, to increase rapidly in violence, without exposing the patient to very serious danger. Should, therefore, the high-toned fever, usually attendant on severe labours, not subside in the course of a few hours after a delivery with the forceps, the practitioner would have much reason to be anxious about the ultimate consequences of his case; and, at all events, he would have no time to lose in trifling and temporizing practice. On the contrary, he would have to reduce forthwith the excessive vascular action by ample bleeding; to empty the rectum by an oily and demulcent enema; and, as soon as possible afterwards, with a

* For a case of acute mania in which opium was successfully administered in large doses, see the Edinburgh Med. and Surg. Journal for October 1823—R.

view to the further reduction of the constitutional irritation, to place his patient under the full influence of opium; which it would be generally proper to combine with a saline diaphoretic. After causing the vagina to be well cleansed with an injection of warm water, it might be of the greatest use, in many cases, to order the whole of that passage to be charged with a thin, soft linseed-meal poultice. This duty, for an obvious reason, would be best performed by the practitioner himself. The poultice should be withdrawn every six or eight hours, in order to give a free exit to the lochial discharge. On all these occasions, the syringing the vaginal passage with abundance of warm water should not be neglected. I need not observe that due care should be taken not to expose the patient unnecessarily, during the performance of these troublesome, but, to her, most important services, to the action of cold."

We conceive that we do no more than justice to the principles advocated by our author in here publishing the following paragraph, containing statements of the general results to be expected from obstetric operations with instruments, when undertaken too indiscriminately and without a due regard to those invariable principles which ought alone to justify the employment of instruments, as compared or contrasted with the success of our author and others, who must be presumed to bring to the consideration of the subject a thorough knowledge of the art, combined with a proper conviction of the importance of abstaining from the unnecessary employment of instruments, and of acting with the utmost care and tenderness when their aid is unfortunately indispensable.

"In the undertaking and performance of forceps operations, the operator should on no account, even for a moment, suffer himself to forget the comparative value, respectively, of the lives that are entrusted to his disposal. A French writer, of a few years standing, once indulged in some very flippant remarks on the proportions of embryotomy and forceps cases, published by Dr. Bland in his Tabular Reports of the Midwifery Practice of the Westminster Dispensary. The answer is, that in all the forceps cases, or rather vectis cases, of Dr. Bland, the mothers SURVIVED and RECOVERED. I believe the same to be the result, generally, of operations with the forceps, when performed by the more responsible midwifers of this metropolis. In my own practice, as one of the physicians to the Maternity Charity of London, which is, beyond comparison, the most extensive obstetric institution in Europe, I have the satisfaction of being able to assert, that I have never incurred the misfortune of losing a mother in consequence of a forceps operation. This statement is, of course, published deliberately, in the face of abundance of producible evidence to confute it, were it not well founded. But what are the results of forceps operations in the practice of the great obstetric institutions of the French metropolis? Without attaching all the confidence, to which I have no doubt many of them are well entitled, to certain private reports, which from time to time I have received on these interesting topics; I am at least fully entitled to appeal to such documentary evidence, as to the results of the obstetric practice of our ingenious neighbours, as they have themselves thought proper to publish for the perusal of all the world. In a work lately published in France, which professes to give a detailed account of the most interesting cases which have occurred in one of the principal lying-in hospitals of Paris, entitled 'Pratique des Accouchmens, &c. par Madame Lachapelle, sage-femme en chef de la Maison d'Accouchment de Paris,' the author has given particular histories of upwards of ninety forceps cases. Of these more than ninety cases, nineteen are positively stated to have proved fatal to the mother!!!

Dr. Davis asks what can be the motive of the French government in committing the lives of so many of their poor countrywomen so entirely to the hands of incompetent female practitioners, and observes that it surely cannot be right that women should be entrusted with the performance of capital surgical operations, even on other women. We would ask, what becomes of the assertion, that it is needless to bestow particular attention on the study of midwifery; and what are the advantages which may not be expected to result from the attention of the profession in general being awakened, by such works as that of our author, to the importance of adequate preparation on the part of those who are destined hereafter to be entrusted with the practice of that important art?

In the fourth section, Dr. Davis treats of other expedients, besides those already discussed, for preserving the lives both of the mother and her offspring, comprehending the operation for inducing premature labour and the section of the symphysis pubis. With respect to the operation for inducing premature labour, his observations comprehend all that is valuable upon the subject. He concludes them as follows:—

"The operation for inducing premature labour, on account of confinement of pelvis, belongs of right to our first class of instrumental deliveries; it being considered compatible with the preservation of the lives both of the mother and the child. Its proper objects, in reference to this indication, are women having only a moderate confinement of pelvis; its capacity being supposed sufficient for transmitting a child of seven or eight months growth, but too small to admit of a living birth to a child of the ordinary bulk and dimensions at the full period of gestation, even with the assistance of the forceps. When, therefore, a female of such conformation shall have had one or more dead children from this cause, she should become the subject, in her future pregnancies, of this valuable operation. The precise time for its performance should depend on the amount of the supposed deficiency of the capacity of the pelvis. If we suppose the smallest diameter, in any part of the pelvis to be less than three inches, then the operation should be performed in the eighth month; and more or less early in the month, as that diameter is presumed to vary between two inches and three quarters and three inches. On the contrary, if the short diameter is known, or upon good and strong grounds suspected to EXCEED three inches; it might be competent, on the part of the practitioner, to defer the operation till the first or second week in the last month of gestation; and then to perform it in such a way as to insure the accession of labour in the most gradual and natural manner possible."

The peculiar mode of operating here alluded to is described by Dr. Davis as having been performed for the first time 27 years ago by Mr. Jacob Jones of Finsbury-square, and consists in dilating the orifice of the uterus with the finger gradually and by successive attempts, and by that means dislodging the viscid mucus by which nature plugs it up during gestation without perforating the membranes, which latter procedure is, however, to be adopted occasionally in preference to the former, or where it has failed in producing the effect intended.

"The operation for the induction of premature labour," continues our author, " is unquestionably a capital improvement in the obstetric art; inasmuch as it furnishes the means of saving the lives of many children, not only without any increase of risk, but with a great reduction even of the chance of danger to the lives of the mothers. It is in one sense, indeed, truly observed by Dr. Denman, that the objects of the operation are circumscribed within certain limits;—viz. those of a mere sufficiency of space within the pelvis to admit of the transmission of a living child of seven or eight months gestation, but not sufficient to admit of a living birth at the full period."

Dr. Davis here states it as a matter of great satisfaction, that of the entire number of distorted pelves, too small to admit of the birth of living children at the full period, about one third, at least, will be found to be fit for the operation in question.

As to the operation of dividing the symphysis pubis, proposed by M. Sigualt, of Paris, nearly fifty years ago, for the purpose of enlarging the diameter of the pelvis in difficult births, our author remarks, that

" It has been performed with various success in several countries of Europe, from the date of its first promulgation, till within a very short period of the present time. It has been most successful, where, probably, it was perfectly unnecessary; whereas, in cases of actual difficulty, it has seldom answered its proper indication; that of saving the lives both of the mother and her offspring, Where the confinement of the pelvis is considerable, it yields too little additional space to admit of delivery being certainly effected without reduction of the child's bulk, by the further operation of cephalotomy, and when only doubtful, or very moderate, it would often be impossible to come to a positive conclusion as to the expediency of this or any other operation, before it might be too late to ensure the preservation of the child's life. From its great danger even to life, and the deplorable infirmities it has so often entailed upon its subjects, when it has not proved fatal, the Sigualtean operation has now fallen into almost total desuetude. In this country, it has never, I believe, been performed more than once, and in that instance it proved quickly fatal both to mother and child.—*See the London Medical Journal, (Simmons'), vol.* XI. *p.* 46.

The fifth section of our author's work relates to "obstetric operations, calculated to ensure the preservation of the more important life of the mother," and is introduced with the following general observations.

" In cases of so much confinement or distortion of pelvis as to make it incompatible with the birth of a living child by the natural passages, at any period of gestation sufficiently advanced to derive advantage from the operation for the induction of premature labour; the next resource of our art is to reduce the bulk of the child by an operation, or a series of operations necessarily fatal to its life. The head, when naturally proportioned to the rest of the body, is, it is well known, the most bulky part of the child; and, therefore, this is the part which most frequently becomes the object of the distressing operation of which we have now to treat. We have already seen that the fœtal scull consists of several different portions of bone, connected together by softer structure, in such a manner as to admit of its being moulded into diverse forms, and shortened or elongated as to its several diameters by the pressure made upon it by the uterus and other parts concerned in the labour. From the operation of this same pressure the part immediately presenting is put tensely on the stretch; and when deprived of its natural strength and firmness by the previous death of the child, it has been known occasionally to have suffered a spontaneous laceration; the integuments of the head and linings of the scull giving way together; so as to admit of the sudden escape of a part of the brain, and immediately afterwards, of a sufficient collapse of the fœtal head, to render its eventual transmission through the pelvis, by the natural agents of labour mechanically practicable. Inasmuch, however, as this result has only very rarely occurred, and in the circumstances here presumed, both mother and child might generally be expected to perish in the dreadful struggle; it has, for many years, been the established practice of this country, in these most deplorable cases, to ensure the preservation of the more valuable life of the mother, by effecting, artificially, what nature has herself occasionally, though confessedly very unfrequently, been able to accomplish in her own behalf."

The first of the operations usually performed with this view is craniotomy, and for its performance our author prefers a pair of craniotomy scissars like Smellie's, 11 inches in length, $2\frac{7}{8}$ inches from the joint to the points, and $1\frac{5}{8}$ from the stops to the points. The blades are a little curved to admit of their gliding readily along the palm of the hand and conducting fingers of the practitioner. The perforation might be equally well performed with Dr. Denman's perforator, but the scissars, besides serving to perforate the head enable the operator very conveniently to reduce the brain and its involucra into small portions, which cannot be said of the mere perforator. Instead of searching for a suture or fontanelle, Dr. Davis thinks it the safest practice to perforate the presenting part of the head at its centre.

" A sufficient opening being made in the head, a part of the brain may be expected to be forced through it, by the bearing down action of the uterus; and that action continuing vigorous, the child's head will undergo a gradually increasing diminution of its bulk, and eventually, the whole of the fœtal subject will very probably be expelled without any further assistance of art.

" It may here be expected that I should notice a rule of practice, which some authors have founded upon a knowledge of this principle, viz. that of surrendering the required compression and the collapsing together of the child's head to be effected solely by the natural efforts of the uterus and its auxilliary propelling organs; these being considered adequate to the finishing of the labour, even in cases of considerable difficulty, after the previous measure of opening the fœtal head. I beg briefly to state that it is, and always has been my own opinion, that the practice, which this doctrine would lead to, might in many cases be very inconvenient and dangerous. It is seldom that operations of this kind are performed, or ought to be engaged in, until after an advanced period of the labour; until the patient shall have endured considerable sufferings; and until the natural powers of the function shall have been greatly reduced. But in such a state of things, does it seem probable that any substantial advantage could be derived from further delay: provided, indeed, the delivery could be forthwith affected by art, with equal or greater impunity to the structure and functions of the maternal organs? Not to add that protracted labours of the description that we are now considering, become sometimes suddenly complicated with the most dangerous symptoms and incidents; such as require the earliest possible delivery, as an indispensable measure, even to afford a remote chance of saving the patient's life.

" The next step, therefore, in the accomplishment of these untoward labours, is, what has been usually called, from the frequent employment of hooked instruments to effect it, *embryulcia.*"

The learned author next proceeds to speak of extracting instruments for completing the delivery, after the previous use of a perforator, and, alluding to the craniotomy forceps, which he submitted to the profession several years ago, the principal distinctive property of which was the separableness of its blades by a joint like that of the common obstetric forceps, he observes, that the points of the teeth being below the level of the spoon-part of the external blade, it is essential, that the handles be very firmly bound together in order to ensure the adequate penetration of the teeth into the fœtal scalp. Unless this precaution be taken with the craniotomy forceps, the instrument must slip upon the application of even a moderate extracting force.

To obviate such accidents, Dr. Davis now offers, to the choice of the profession, several varieties of crotchets furnished with counterparts, which, while they effectually prevent the crotchet from slipping, are likewise calculated to obviate any risk of wounding or lacerating either the maternal structures, or the hand of the operator, accidents which are frequently unavoidable with the common crotchet, when any considerable force is applied, even by the most dexterous hands. The crotchet blade of one of these new instruments, which are very properly named *guarded crotchets*, is furnished with a broad hook, which is divided into three sharp and strong prongs, each about half an inch in length. This blade is to be conducted into the vagina, and along the fingers of one hand previously introduced, so as to apply the prongs to the part of the scalp which it is intended they should penetrate. The other blade or guard is then to be introduced within the cranium, through the aperture previously made by the perforator. Upon adjusting the two counterparts at the lock, and firmly compressing the handles, the prongs enter the scalp, and upon moderate extracting force being applied, they will even penetrate through the scull. The handles may then be secured, or not, with a tape, at the pleasure of the operator, this precaution not being so essential as in using the craniotomy forceps.

This instrument, the construction and application of which is extremely well illustrated by a plate, seems so admirably calculated for the object proposed, that it might be deemed almost superfluous to have any other for the same purpose; but, as the instrument just described is adapted for perforating the scull from the outside, and as it has been generally the practice in this country to fix the crotchet into the scull from within its cavity, our author's inventive talent has enabled him to furnish an instrument to be applied in that manner, without giving up the security of an effectual guard. This variety of guarded crotchet is also faithfully represented in a beautiful plate, and seems perfectly well calculated for the intended purpose. The author justly observes of these guarded crotchets, that while their construction is such as to prevent sudden lacerations of the fœtal scalp or extensive fractures of the bones, their power can only act, during every stage of their use, in perfect conformity with the intention of the operator. Dr. Davis mentions that some of his friends have considered these crotchets as unnecessarily bulky. His answer to this objection is, that the strength of the instrument is no farther an objection than can arise from its weight. The precise weight of one of the first described variety of crotchets is one pound and five ounces avoirdupois. The object of so much power is, to provide against the possibility of any accidents which might otherwise arise from the easy bending or mere action of the elasticity of either of its branches. The first principle of all extracting instruments, with sharp hooks or teeth, should be an inflexible firmness of purchase. With such an instrument more power may be used in a given time, and compatibly with perfect safety, than with any hooked instrument without a guard, which cannot, moreover, be used without great risk. Not only may more abstract force be applied, but the amount and direction of it may be varied to a considerable extent without losing or letting slip the hold of the instrument, while any attempt to vary the direction of the extracting force with a common crotchet would almost certainly cause it to slip.

We here subjoin some of the "rules and precautions" laid down by our author as "necessary to be observed in the use of craniotomy instruments."

"1. The operation of craniotomy, necessarily destructive to the fœtus, can never be thought of as a justifiable measure, until all hope shall have been extinguished, of being able to accomplish the birth of a living child by the natural passages, compatibly with a satisfactory degree of certainty of the mother's recovery.

"2. To ensure the safety of the mother's life, it is to be presumed that the soft parts must not be exposed to the risk of dangerous contusion, from the violence or long duration of pressure upon them of any kind, whether occasioned by the use of forceps or other implements of our art unsuccessfully applied.

"3. In cases of doubt as to the eventual competency of the natural powers to effect the delivery, or at least to propel the head of the child sufficiently into the pelvis, to be within the safe reach of the forceps, the benefit of that doubt should always be given in favour of time; so long at least as it may be compatible with the absolute safety of the mother to put off the moment of interference. In all estimates, however, of the probable results of doubtful cases, the practitioner should never lose sight of the important fact, which, in this Protestant Country, is not disputed, that the life of the mother is incomparably more valuable than that of the child.

"4. Before we yield finally to the suggestions of a professional expediency so deplorable as that of having recourse to this fatal operation, it cannot be for a moment doubted that we should hold ourselves morally bound to avail ourselves of all other less formidable resources of our art. Under this remark I may perhaps be permitted to recommend, as an essential preliminary measure, a very cautious trial of the long forceps.

"5. The safe and proper use of the long forceps requires, however, as we have already seen, the union of much dexterity and intelligence. It is a power to be tried for the benefit, essentially, of the child; but not at the expence even of risk of additional danger to the mother. It may be claimed as a merit of the forceps, with blades of unequal length, that they are of a form peculiarly calculated to enable the practitioner to apply to the child's head a considerable amount of attracting force, without necessarily exposing the parts at the brim and within the cavity of the pelvis to the risk of injurious contusion.

"6. In cases of great confinement at the brim of the pelvis, and the gestation being presumed to have arrived at its full period, the practitioner may avail himself of some latitude as to his choice of the time for making the aperture in the fœtal scull. In the case, for instance, of a pelvis having for its conjugate diameter any measure short of two inches and a half, and that fact being assumed to have been ascertained positively and beyond the possibility of doubt, by accurate admeasurement, he might then be permitted to introduce his perforator at almost any period of the labour; but generally the sooner the better after the rupture of the membranes, and after the accession of sufficient development of the orifice of the uterus, to ensure the safety of the operation. The rule which has been commonly prescribed on this point, is that of not perforating the head until the orifice of the uterus shall have become widely dilated. In cases, however, of so much confinement of the pelvis as we are now supposing, I have never known an instance where the orifice of the uterus became, at any time, dilated to the extent of its full capacity of development in ordinary circumstances. It should be considered that, as the head of the child, in these cases, cannot effect its entry into the pelvis, by reason of its defective capacity, this natural instrument of dilatation, viz. the fœtal head, is not in a situation to exert its usual and properly dilating power, upon the uterine orifice."

The seventh rule directs the extracting force to be applied in the direction most agreeable to the mechanism of the birth, that is, as much as possible in correspondence with the long axis of the fœtal head; and strictly in a line, during every stage of the extraction, with the curved axis of the pelvis. It enjoins the immediate and careful removal of any fragments of bones which might injure the vagina if heedlessly extracted, and recommends a change of the situation of the crotchet to some other part of the scull, in the event of several well directed efforts being made without apparent effect.

"8. It is a very good rule, to be attended to in all cases of operative midwifery, where the application of artificial force is indicated, that the force to be used should be applied, in the first instance, in a very moderate degree, which then should be progressively, but still most cautiously, augmented according to the demands of the particular case. It is an advantage, nearly equal, of both modifications of crotchets with guards, which have been described, that they are compatible with the exertion of a degree of force greater beyond comparison than can be exerted with equal safety with any kind of unguarded crotchet. In perusing this statement, however, the reader must not suppose that any degree of attracting force, to be limited only by the operator's muscular power, may be used with impunity in these operations; for it may be easily understood that a powerful man might be competent, in point of physical strength, to divide in sunder the entire pelvis of his unfortunate patient, with any sort of instrument that might ensure for him an unyielding purchase. What is intended to be conveyed is, that much greater force may be used WITH IMPUNITY with a guarded, than with an unguarded crotchet; and, indeed, the utmost amount of force that it can ever be safe in such cases to apply. With a guarded crotchet the practitioner is at liberty to use both his hands at the same time; by which he becomes competent to effect his attraction with much more steadiness and accuracy, as also to vary the direction of the force applied, and the bearing of the purchasing part on the fœtal head with a considerable accession of effect."

The ninth and last rule relates to cases of preternatural labours, in which, on account of confinement of the pelvis, the head is detained at the brim after the delivery of the body. Our author expresses his opinion that, in such cases, the child might be more frequently brought alive into the world, by passing up two fingers of one hand into the child's mouth, applying the other hand to the shoulders, and drawing down in a line with the axis of the brim, than by any kind of forceps whatever. But in cases either of great confinement of the pelvis, or morbid enlargement of the head, rendering delivery by the hand impracticable, he justly observes, that the life of the child must soon cease to be an object of protective treatment, and, after its death, there could no longer be a question as to the propriety of opening the head in preference to the application of much extracting force, without craniotomy, whether that force were exerted by the hands alone, or by the forceps. The perforator, in such a case, is to be made to enter the scull immediately behind the more accessible ear, and when the contents of the scull are discharged, there will generally be no other difficulty in extracting the head.

The varieties of crotchets already described, are applicable solely to the heads of children, but it has sometimes happened in cases of great confinement of the pelvis, that, even after the complete removal of the child's head, its body has remained above the superior distorted aperture for many hours, and in defiance of repeated attempts to extract it. Our author has given in the plates beautiful representations, (which are accompanied with suitable instructions,) of two varieties of guarded crotchets for this purpose, under the designation of *body crotchets.*

In cases of embryotomy on account of great distortion, as where the conjugate diameter of the pelvis is under two inches or two inches and a half, it will be with extreme difficulty to the operator and much danger of injury from contusion of the maternal parts, that a full grown child can be extracted piece-meal, with the assistance even of the guarded crotchets which are described by Dr. Davis.

"After all," says he "there must be limits somewhere to the safe use of crotchets, and these limits ought certainly to be such, as should exclude even an approach to a violent exertion of attracting force; which might compromise the security of the important ligaments of the pelvis, or, if possible, the other still more important structures contained within its cavity.

"In urging professional attention to the careful observance of these limits, I am happy to feel myself in a situation to offer a safe substitute for the exertion of inordinate force in the treatment of these deplorable cases; not only without contracting the limits of our art, but compatibly with a considerable extension of its power. The expedient I allude to consists in the application of a simple, but very effectual contrivance, for effecting a much greater reduction of the fœtal scull than has hitherto been attempted in the practice of modern times. It is, indeed, a power, by which any portion of the fœtal skeleton, presenting at the brim of a contracted pelvis, may be broken down into small fragments of about half an inch in diameter, with the most perfect impunity to the structure of the parts of the mother concerned in the operation."

Representations of this very ingenious, but simple instrument, are given in the plates, under the designation of osteotomist or bone pliers.

"As far as I know," continues our author, "it is an example of a new application of mechanical power, combining the principles of a punch and a pair of scissars. The whole instrument is made of solid and well-tempered steel. Its cutting ends are worked into two long and fenestrated oval rims of unequal size, but of nearly equal strength. The smaller is of a size to enter into, and to fit closely within the parietes of the larger. The mutually adapted parts of each, being formed into a continuous oval edge, they become competent, when brought together and firmly applied to their object, to exert a prodigious power upon a portion of bone placed within their grasp. The handles are of great length in proportion to the parts anterior to the joint; and being of sufficient strength to be perfectly inelastic and inflexible, their power must be deemed equal to the full length of their leverage, multiplied by the muscular force employed in using them.

"I have often, out of curiosity, and to show the extraordinary power of this instrument to my pupils, made breaches into strong ribs of beef, by cutting out of them a succession of pieces. Having a power like this in reserve, it is evident that the employment of any inordinate force of attraction with the crotchet may, in almost all cases whatever, be happily and certainly avoided. One or two sections taken out by the osteotomist from the basis of the scull, which is by far the most bulky part of the fœtal cranium, will generally have the effect of putting an end to all difficulty. In cases of greater confinement, a few additional sections will, perhaps, be required to be made, in order to give a sufficient degree of facility to the afterpart of the operation."

The extreme breadth of the broadest oval rim of the osteotomist is precisely three quarters of an inch. It may, therefore, be taken for granted, that wherever there may be sufficient space to admit of the introduction of this instrument, together with the point of an index finger to feed it with successive purchases of bone, it will be practicable to effect, and therefore prudent to attempt the delivery by the natural passages. There are few pelves, even in large collections of distorted ones, with superior apertures so small as not to furnish from between an inch and an inch and a half of space in the direction of their conjugate diameters; or, at least, of antero-posterior diameters across some part of their brim. In any such cases, our author says that he would think it his duty to avail himself of the use of the osteotomist, and to undertake delivery by the natural passages; and from experiments, that have been again and again repeated, with dead children upon artificial distorted pelves in Dr. Davis's theatre, we are warranted in concluding that, in Dr. Davis's hands, the osteotomist would, in such cases as he has above supposed, certainly ensure the safe delivery of the patient.

Persuaded, as we are, that, with proper care in the hands of an intelligent practitioner, the osteotomist is capable of rendering delivery as easy and safe to the mother in cases of extreme confinement of the pelvis, as delivery usually is with crotchets after craniotomy in cases where the distortion is not so considerable, we have no hesitation in declaring our opinion, that the invention of the osteotomist is alone sufficient to entitle its author to the thanks and respect of the profession, and to the gratitude of the unfortunate beings for whose benefit it is intended.

Our author concludes his observations on the osteotomist as follows:

"If, indeed, I am not greatly overvaluing the power of this instrument, it will not only enable skilful operators to effect deliveries in cases of moderate distortions with much more facility to themselves, and proportionally less danger to their patients than heretofore; but it will also have the effect of reducing, almost to zero, the necessity for having recourse to that last extremity of our art, and the forlorn hope of the unhappy patient, the Cæsarean operation. In this country, it is well known that, with one exception, (for I see no reason for disputing Mr. Barlow's case,) the Cæsarean section has uniformly failed in the more important part of its object; that of preserving the most valuable life of the mother: whilst in France and Germany, where it has been most frequently performed, its fatality, however variously reported by its friends and foes, has been universally acknowledged to have greatly exceeded in frequency its happy results. Any suggestion, therefore, for superseding the necessity of so formidable an operation, or even for greatly reducing the frequency of that necessity, seems entitled to the attention of the profession; and that, indeed, is all that I have at present a right to claim in favour of the osteotomist."

With such an instrument at hand, an operator is certainly and happily relieved of all motive, and also deprived of all excuse for obstinate perseverance in attempting to deliver his patient by main force, whether by means of crotchets, or by any other simply extracting instrument whatsoever. In order to use the osteotomist with safety and effect, the whole of the left hand should be introduced into the vagina. The index and long finger must then be carried forward into distinct contact with the portion or fragment of cranial bone most immediately presenting, so as to be able to feed the mouth part of the instrument with successive purchases, and to guard effectually every part of the maternal structure from entering within its grasp. For more particular directions, and cautions, to be observed in using the osteotomist we must refer to the work itself.

With reference to the smallest space at the brim of the pelvis, with which delivery by the natural passages is practicable, compatibly with the patient's life, Dr. Davis makes the following observations:

"Among my machines for instructing pupils in the treatment of difficult labours of this class, there is one constructed on the model of Elizabeth Thompson's pelvis. Dr. Hull, in concluding his description of the dimensions of the brim of that pelvis, observes, that 'the largest circle that can be formed in any part of the superior aperture does not exceed in diameter one inch.'—*See Hull's Observations on Mr. Simmons' Detection, p.* 197. The machine constructed on this model, which is the smallest, as to the capacity of the pelvis, of ten specimens of distortion affecting this part, taken partly from actual subjects in my possession, and partly from published descriptions, is made, like all the others, of very firm wood, the heart of oak. The distorted cavity was carved out accurately to the dimensions given in the above history, from the solid timber. An os coccygis, made of pretty strong leather, and appended to the part of the apparatus representing the sacrum, as also sacro-ischiatic ligaments, of the same materials, and certainly not less (quære, more) yielding than their natural prototypes, are fixed, at their proper points of attachment respectively, to the sacral and ischial bones, by strong brass plates. At the distorted brim, there are fastened three pairs of powerful leather straps, such as saddlers use; of which, one of each pair is tipped with a strong buckle, and the other is perforated, at half-inch distances, with holes. The parietes of the superior apertures shelve laterally into the costal surfaces of each iliac bone, and posteriorly into the lumbar vertebræ. To exercise the pupils in this very important branch of operative midwifery, it has, for several years, been the practice of my school, to provide for them occasional opportunities of performing embryotomy operations on real fœtal subjects. For these purposes, the little subjects, generally the produce of still-births, or of deaths supervening shortly after birth, are firmly strapped down, in the position determined upon, at the brim of distorted pelvis, and a principal operator and two assistants are appointed (usually by ballot) to undertake the delivery. A pelvis of only moderate defect of capacity is, for the most part, selected for the first operation. The proper instruments are then given out, and the operation of embryotomy is duly undertaken and performed, with strict attention to all its ordinary circumstances and stages; and the extraction of the child, by reason of the moderate amount of the deformity of the pelvis chosen for the first operation, is, for the most part, effected without much difficulty. The representative of a more distorted pelvis is then fixed, as in the former case, upon the pedestal. The fœtal subject, already excerebrated, and possibly otherwise damaged, is strapped down, most frequently in a different position from the former, at the superior aperture of the pelvis, and other operators are appointed to effect the extraction.

"The difficulty of this second operation is such as very greatly to exceed that of the former; and may generally be presumed to bear a pretty direct proportion to the greater distortion of the pelvis chosen for the experiment, added possibly, in some cases, to a little want of dexterity on the part of the young operators. After this preliminary account, the reader will be able to affix a proper estimate to the statement about to be made as to the smallest probable space within the pelvis, which might admit of an embryotomy delivery by the natural passages; the practitioner being supposed to have the option of using the osteotomist. The fact to be stated is shortly this, viz: that several fœtal bodies, of the ordinary size of children at the full period of gestation, I am not sure whether four or five, and all, with one exception, damaged by previous operations, have, at different times, been the subjects of successful attempts to effect their transmission through the wooden pelvis which has been described as a perfect representative, *as to its dimensions*, of the pelvis of Elizabeth Thompson. In the one exception just adverted to, the fœtal subject was strapped down to the machine *perfectly whole*, for the special intention of deciding the fact of the practicability of its delivery through such a pelvis, in the absence of circumstances which might be considered as unfair advantages. In all these experimental exercises, the extraction of the fœtal subject was finally accomplished; sometimes, indeed, with more or less of difficulty, but in one instance, I well remember, with a considerable degree of facility."

In these operations, no violent extracting force was permitted to be used, but the bones, and especially those of the fœtal head, were removed with all possible care, in successive portions, by the osteotomist. Dr. Davis candidly apprises his readers that the machines in question are mere representatives of deformed pelves, without any soft parts contained within or appended to them, and proceeds to give ample and clear directions as to every step of the operation, from which we think we shall be justified to our readers, by the importance of the subject, in extracting what follows.

"The danger fairly imputable to an embryotomy operation, would be exclusively what might be conceived to arise from lacerations or contusions, occasioned either by the hands of the practitioner or by his instruments. I have already stated that injuries of that kind need not necessarily be inflicted. It would undoubtedly be competent, on the part of the practitioner, and, at the same time, his bounden duty, in every instance, to avoid them. Impatience should never be allowed to quicken his movements; and even the endurance of pain or fatigue, from protracted support of the same attitude, or from great pressure on his hand and fingers, incident to the performance of such duties, should arm him rather with additional motives to be temperate and cautious in every stage of the procedure. The object most to be considered, is not whether the operator's fingers should be engaged at the brim of the pelvis for any particular time, that is, whether for one or three hours; but whether it is permitted, when there, to act violently or forcibly during the whole or any part of the time it may be so employed. The use of instruments in the same situation is obviously subject to the application of a precisely similar remark. It may be of little or no consequence, for instance, that the osteotomist should be engaged at the brim of the pelvis, performing its retired duties slowly and safely for a considerable duration of time, and of which it would scarcely be prudent to prescribe the limits, provided it were perfectly well guarded, by the practitioner's fingers, from pressing injuriously on the soft parts, which otherwise would be especially exposed to dangerous contusion."

The minute and particular instructions laid down by our author for the use of the osteotomist, are followed by some excellent and appropriate observations on embryotomy operations in general, comprehending a consideration of those cases in which it becomes necessary to evacuate the cavities of the thorax and abdomen of the fœtus, or otherwise to mutilate its body. We extract the following part of what relates to the comparative merits of the operations of turning and embryotomy, in the management of cross presentations, in certain very untoward circumstances which occasionally perplex the most experienced midwifers.

"Let it be a presumed fact, or even a matter of strong suspicion, that the uterus and bladder, peritoneum, blood-vessels, or any other structures situated at or in the neighbourhood of the brim of the pelvis, were in a state of contusion from the long duration and great violence of the parturient action; but still that the action in question might not be quite extinct. My professional experience has supplied me with several opportunities of knowing that the womb has been ruptured by violent and pertinacious attempts to perform the operation of turning in these untoward circumstances. A state of contusion is, in other words, a condition of morbid tenderness of the structure of any part so affected. It can never be supposed to exist without being accompanied by symptoms of inflammatory action, indicative of a progress more or less rapid towards an eventual exhaustion, both of the uterine and constitutional powers. The patient's life may be presumed to be already in a state of no little jeopardy; and great indeed, in my opinion, would be the hardihood of that practitioner who would thrust forcibly into the cavity of the uterus so large an instrument as his hand and arm, in circumstances so totally devoid of a prospect of being able to accomplish any good by it. From such an operation I scarcely need observe, there would be every thing to fear and scarcely any thing to hope for. In all human probability the child would be dead-born, if not actually putrid; and the mother's life, already in some peril, would be involved in tenfold danger.

"The expediency of the operation is surely not to be measured by the competency of a practitioner, in point of muscular strength, to accomplish it; but by a fair estimate of its promise and adequacy as a means of saving the child's life, without exposing that of the mother to much additional danger. If, therefore, we suppose the child to be already dead, or the circumstances of the labour to be such as to make it impracticable to bring it into the world alive by means of turning, or even to perform that important operation at all, without exposing the mother to extreme danger; it would then, in my opinion, become the unquestionable duty of the practitioner to effect the delivery by embryotomy. The child, in the case supposed, being situated transversely, the first step of that procedure would be, to divide it into two principal parts, head and body, by passing a properly adapted instrument across and through the entire structure of the neck. Before the introduction, by Ambrose Paré, of the modern operation of turning by the feet, it seems probable that the detruncation of the fœtal subject was occasionally performed by the older surgeons, and, perhaps, more frequently than it may have been thought of since that period. The separation of the child's head from its body, in the very circumstances of which we are now speaking, was, indeed, very distinctly recommended by Celsus:—'Si vero transversus est, neque dirigi potuit uncus alæ injiciendus, paulatimque attrahendus est. Sub quo fere cervix replicatur retroque caput ad reliquum corpus spectat. Remedio est *cervix præcisa;* ut separatim utraque pars auferatur. Id unco fit qui priôri similis, in interiore tantum parte, per totam aciem exacuitur. Tum id agendum est, ut ante caput, deinde reliqua pars auferatur: quia fere, majore parte extracta, caput in vacuam vulvam prolabitur extrahique sine summo periculo non potest.'—*Celsus, lib.* vii. *cap.* 29.

"It seems, however, certain, that this idea of severing the fœtal head from its body has never obtained a very general prevalence among practitioners in midwifery. Heister, indeed, alludes to it as an operation newly invented in his time by Van Hoorn.—*Heister Institut. Chirurg., cap.* 153, *sect.* 9. The best recorded case of this kind that I am acquainted with, is one which was published some years ago, by Dr. Sims, in the Medical and Physical Journal. It cannot, I think, be disputed, that the general principle of the operation is just; and I am rather surprised that its almost absolute indispensableness, as a means of saving the mother's life, has not long ago, and more especially since the publication of Dr. Sims's paper, given it the importance of an established rule of practice in the treatment of arm and shoulder presentation cases, *not admitting of the safe performance of the operation of turning.* So far, however, is this from being the case, that the art has not even yet possessed a safe and suitable instrument to meet the proposed indication."

To supply this defect, our author presents the profession with a choice of two instruments, one of which is named the *guarded embryotomy knife*, and consists of two counterparts, fitted with a joint like that of the common forceps. One of the blades is armed with a knife, diagonally attached to its shank, and hook-like point. This blade being introduced at one side of the child's neck, and the other blade, which merely serves as a fulcrum and guard, at the opposite side of the neck, the handles of both are to be adjusted at the lock, and then, while the operator is careful to ascertain, with the fingers of one hand, that the instrument is properly applied to the neck, he may, with moderate extracting force steadily applied with the other hand, effect the decollation of the fœtus, without any risk whatever of injuring the mother.

The other instrument for effecting the same purpose, seems to be very much the same as that alluded to by Celsus in the above quotation, namely, a common blunt hook, with a sharp edge on the concave aspect of its curve. Dr. Garthshore once divided the neck of a putrid child, while he was attempting to extract it with a blunt hook applied to the neck, and Dr. Sims, in a similar case, found the ligaments so firm, as to permit the child to be extracted twofold without giving way. This suggested to him the having a blunt hook, with the inner side of the bend filed to an edge, to be used in such cases. Dr. Ramsbotham has been in the habit of using such an instrument, and has allowed our author to have a drawing taken from it.

When the decapitation is effected by either of these instruments, a very moderate extracting force, applied to the protruded arm, will bring out the body of the child. The head may afterwards be extracted with forceps, if such assistance be required, or it may be perforated with the craniotomy scissars and extracted with the guarded crotchet. Dr. Davis once met with a case where the head was lodged very high up within the contracted uterus, and where it appeared that the perforation of it might have been facilitated by the aid of a pair of craniotomy scissars of greater length than those in common use. He thinks that one of twelve inches and a half would have been required to meet the difficulties of the case.

In some deplorable cases of great distortion and confinement of the pelvis, our author justly observes, that it would require the most deliberate consideration of a practitioner, and the assistance, if attainable, of a consultation, to determine whether the Cæsarean operation might not deserve the preference to any mode of delivery by mutilation of the child; as the latter might expose the mother to so much additional contusion and friction of the parts concerned, as could scarcely fail to prove fatal. The reasons for and against resorting to this last and melancholy resource of the obstetric art, are weighed by the author with his usual ability and perspicuity, and will be found highly deserving of careful perusal.

Before concluding our account of the important work before us, we cannot refrain from expressing the conviction we feel, that it is calculated to have a most beneficial and lasting influence. By bringing the just pretensions of midwifery, as a science, and its undeniable resources, as an art, in a clear and convincing manner before the profession, and by the manly and spirited language in which the learned author has asserted its claims, as a legitimate and interesting branch of medical knowledge, we have no doubt whatever, that a most useful impulse must be imparted to its pursuit, and we are confident, that a great deal has been done towards dispelling the pernicious cloud of prejudice, that has too long overshadowed the study, and retarded the improvement of this valuable art. With respect to the probable influence of this publication, in relation to the reputation of its author, we are decidedly of opinion that the Elements of Operative Midwifery, as the production of a happy combination of original talents, deep research, unremitting industry and extraordinary ingenuity, cannot fail to hand down the name of Dr. Davis to a remote period, as one whose zealous and well-directed exertions shall have mainly contributed to rescue this most useful department of science from unmerited obloquy, and to elevate the practice of midwifery, in the estimation of the profession in general, to a level with the kindred branches of medicine. We take leave of the author with the most sincere wish, that he may long live to see the principles he has so ably inculcated, and the instruments he has so effectually improved, honored with the approbation of his professional brethren; and, as he must already possess the conscious satisfaction, of having, in this work, discharged his duty towards the profession, in a manner worthy of the subject and of his own reputation, so we feel assured, that he shall likewise in due time obtain, in the remuneration of an extended and lucrative practice, the appropriate and well-earned reward of a service so essentially beneficial to the community.

Before quitting the subject of midwifery, we think it our duty to advert once more to the defects of the present system of medical education in this country, so far as relates to this important department, and to the deplorable ignorance of some, who are in consequence permitted to engage in its practice. We were led to reflect on this part of the subject, by the general tenor of the observations contained in the preliminary section of Dr. Davis's work, but we were more particularly struck with its importance, upon perusing one of the rules formally laid down by him in discussing the use of extracting instruments. The rule alluded to is as follows: "A hooked instrument should never be employed as a substitute for the forceps or vectis, in cases of head presentation. This observation is meant, of course, to imply that the case to be treated is one of a character not to require the sacrifice of the child's life, as an essential condition to the preservation of that of the mother." The moral obligation of such a rule is so self-evident, that we could not but regard it as superfluous, when addressed to the medical profession in this country and in the present age. Our surprise was not lessened, as our readers may well suppose, by the perusal of the following cases which suggested, to our author, the necessity for the rule in question.

"In a case of natural but tedious labour, it was deemed advisable to have recourse to the use of the forceps, more, however, with a view to accelerate, in some degree, the progress of the function, than to accomplish the whole of the delivery. Accordingly, when the fœtal head had been brought down to bear pretty strongly on the perinæum, the instrument was withdrawn, and the gentleman in attendance was positively assured, that the child would very certainly be born in the course of a few hours. At an early hour on the following day, I was, however, informed that the child had been delivered with instruments, in about two hours after I had left the patient, but that it was still-born. This information greatly surprised me; and, upon learning that the child's head had been wounded, I requested permission to see it, as I concluded that the usual operation of craniotomy had been performed upon it. That, however, was not the case; but a sharp, hooked instrument (no doubt the common crotchet) had been applied to the occipital scalp, without any previous use of the perforator!

"In the year 1820, a poor woman residing in a market town in Staffordshire was attended in her confinement by a Fellow of the Royal College of Surgeons. The labour continuing longer than was quite agreeable to the medical attendant, that extraordinary Member of the Royal College determined to have recourse to the use of instruments; and, not having his forceps at hand, (if, indeed, he ever possessed a pair of forceps,) he took a pot-hook out of the fire-place, introduced it into the uterus of his patient, seized hold of the child's head, and so extracted it. On examination after death (for of course the poor woman died,) it was found that the pot-hook, which had entered one of the orbits, and escaped at the frontal suture of the fœtal cranium, had penetrated the mother's uterus, and lacerated the vagina from top to bottom."

In giving a place here to these disgraceful but authentic cases of ignorance and inhuman conduct, and in the observations which we are about to make, we beg leave to disclaim all intention of arrogating to ourselves any right to censure the proceedings of any public body, and we would at the same time deprecate the idea of being mistaken for conceited, hot-brained reformers, who can discern nothing commendable in existing establishments. Our object is simply to call the attention of the profession to certain facts, which are sufficiently notorious, and which, in our opinion, demonstrate that the present custom, which permits men to enter into practice as accoucheurs, without having been subjected to any examination, as a test of their competency, is as discreditable to the medical profession, as it is prejudicial to the interests of the public; and to subjoin, as hints for general consideration, the outlines of a plan, the adoption of some modification of which, appears to be conformable to the intelligence and spirit of the age, and essentially required by the wants of the community.

It is generally known, we believe, that neither the Royal College of Physicians nor the Royal College of Surgeons deem it necessary to enquire into the competency of their members to practise midwifery, yet it is notorious, that gentlemen, who have obtained the honor of diplomas from these learned bodies, often engage in the practice of midwifery, and the majority, we doubt not, acquit themselves creditably in the discharge of its duties: but one, at least, of the cases detailed above shews, that a diploma from the College of Surgeons is no proof of competency for the practice of midwifery. Indeed, it would be a libel upon the good sense of both the Royal Colleges to infer, because they do not enquire into the fitness of their members for that practice, that they are of opinion, that every man, who has obtained a diploma from them, must be competent to practise midwifery, even though he may never have attended a single course of lectures, nor bestowed any pains on the study of it, nor undergone any examination whatever, as a test of his acquaintance with the subject. To impute such an opinion to these learned bodies, would be equivalent to saying, that they are capable of countenancing those worthless men, who, without any particular study or preparation for the duties of that department, are bold enough, under pretence of performing obstetric operations, to perpetrate acts disgraceful to the medical profession and revolting to humanity.

Is it right then and proper, or is it consistent either with the honor of the medical profession, or with the public good, that any person, whether he may have obtained a diploma from either of the Royal Colleges or not, should be at liberty to engage in the practice of midwifery, and to undertake, at his own uncontrolled discretion, the performance of capital operations, merely because the Royal Colleges do not deem it necessary to enquire into the fitness of their members for such peculiar duties? To this question the two lamentable cases detailed above furnish the appropriate reply, in an emphatic negative, from which, we cannot believe, that one voice in the whole profession will be found to dissent. Should there, however, be any individual, belonging to the profession, so little aware of the peculiar and very responsible nature of the duties of a practitioner in midwifery, as to maintain, because it is a branch of surgery, that its ordinary duties ought always to have been wholly confined to the female sex, and that it ought never to have been tolerated as a line of practice for men, we would at once reply, that such notions are repugnant to the general sense of the medical profession, as well as to that of the female sex themselves, as evinced by the customs of those civilized nations, where the cultivation of the art of medicine has been carried to the greatest perfection. We would ask those who allow themselves to speak of the practice of midwifery with disdain, whether they would see their wives or daughters perish in child-bed, and not apply for assistance to gentlemen of eminence in that department, or, whether, for the sake of consistency, they would prefer, on such an occasion, the assistance of Sir Astley Cooper or Mr. Abernethy, to that of Dr. Davis or Dr. Gooch, or the services of an illiterate female to either of those gentlemen. If those, who have lately taken upon themselves to promulgate such extravagant notions, be really in earnest, we would advise them not to commit themselves again to print upon any subject, without previously obtaining some information respecting it. Certain we are, at least, that the most respectable and best informed members, of the Colleges of Physicians and Surgeons, would not choose to practise midwifery without due preparation and study of its peculiar duties, and that the most eminent and distinguished among them would not hesitate to give a practical proof of their opinions, that midwifery is properly entitled to be established as a separate department of practice, by declining, for example, to sanction, or to perform the Cæsarean operation, unless it were previously declared to be necessary, by a gentleman conversant with the practice of midwifery, in any case where it might be practicable to obtain such opinion.

Assuming then, that it is necessary and proper, that no person, whether a member of the Colleges of Physicians and Surgeons, or not, ought to be permitted to engage in the practice of midwifery, without having passed an examination as to his competency for the peculiar duties of that department, the next question to be considered is, how this competency is to be ascertained? Upon this point we may perhaps be allowed to express our sentiments with the less reserve, since both the Colleges of Physicians and Surgeons disclaim all responsibility upon the subject. We are, then, firmly persuaded, that the interests of midwifery would not be advanced, by these learned bodies either spontaneously assuming, or being invested by the Legislature, with authority to examine candidates and grant licences or diplomas for the practice of midwifery. We might refer to the errors which proverbially abound in the London Pharmacopœia, as a proof of the inexpediency of confiding to numerous, though learned bodies of men, the superintendance of a department, with the practical duties of which they are not habitually acquainted, and, as showing, that though they may be ever so well versed, from reading and private study, with the principles or theory of an art, it is not to be expected, that they should be exempt from error, when they undertake to direct its practical details: a scientific knowledge of words or names not being quite equivalent, to an habitual acquaintance with the things they denote. For the sake of the art itself, therefore, we should regret, to see the duty of examining candidates for midwifery, entrusted to any but the most distinguished professors in that department, whose daily habits of intimacy with its details would ensure the correctness of their decisions, and unquestionably prevent incompetent individuals from being permitted to engage in practice. In our humble opinion it would be an act worthy of the Government, or of the Legislature, to establish a tribunal, whether under the name of Commission, Board, or College, for the special purpose of examining candidates, and granting licences or diplomas for practising midwifery.

In expressing this opinion we would not be understood, as desiring to see midwifery erected into a profession or art, detached from the general study of medicine. On the contrary, it might deserve consideration, whether it should not be an established rule, that no persons' name should be placed upon the list of candidates for examination, except Members of the Colleges of Physicians and Surgeons of London, Edinburgh, and Dublin, graduates in medicine, or gentlemen who had previously passed examinations, and obtained licences to practise as apothecaries. If it should be deemed, in any degree, incompatible with the honor, or trenching upon the privileges of the Royal Colleges, to require that their members should submit to an examination, let them either with-hold their diplomas, until candidates can produce certificates from the Board of Midwifery, of their fitness for practice in that department, or restrict their members from practising midwifery altogether.—Regulations to this effect would certainly prevent the recurrence of such cases of malpractice as those detailed above, which, without any straining of the terms of one of the bye-laws of the Royal College of Surgeons, may surely be deemed "derogatory to the honour of the College, and disgraceful to the profession of surgery." At least, should any such cases afterwards occur, the Royal Colleges would be free from any stigma of that kind that must more or less attach to them, while their diplomas are used with impunity, by profligate and daring men, as a cloak or sanction to their engaging in the practice of midwifery, without adequate preparation. But, whether the examinations of candidates for midwifery should be instituted before, or after they may have obtained diplomas from the Royal Colleges, we regard midwifery merely as a branch of medicine, too important, indeed, even for those, who may have been regularly educated for other departments, to be permitted to practise it without due preparation and examination, but still essentially a branch, and, therefore, we should deem any arrangement decidedly objectionable, that might have a tendency to admit any, but men of regular medical education, to pursue it as a profession.

To ensure, in the most effectual manner, the object proposed by the appointment of a Board for the examination of candidates for the practice of midwifery, there are some circumstances that have occurred to us as of great importance to be attended to, which we shall take the liberty of submitting for the consideration of our professional brethren. The most important of the circumstances, to which we allude, relates to the mode of conducting the examinations. We conceive that many advantages, which could not be so well attained by any private examination whatever, would be secured by the examinations being conducted publicly, that is to say in some hall or theatre, to which the professional public, at least, should have free and unrestricted access: one advantage of this public procedure would be a sedulous and diligent attention to their studies, on the part of gentlemen intending to become candidates. It would be, to the examiners, the best possible protection against the odium that malevolence might attach to the purest characters, if they should have occasion, as members of a public Board, to pass, in secret conclave, their own or each other's pupils. It would probably put an end to the custom of spurious or pretended teachers, if any such there be, purchasing a transient publicity for their names, by advertising lectures on midwifery, which they do not deliver, while it would ensure the utmost exertion, to the faithful discharge of their duties, on the part of those actually engaged in the business of teaching midwifery; as the proficiency of candidates, at the public examinations, would always be looked upon, in a certain degree, as a test of the ability of their respective masters.

We would farther suggest, that it should not be imperative on a candidate, to produce tickets or certificates, for any specified number of courses of lectures, on the subject of the examination, to which he is to submit. The examiners should be bound to ascertain the extent of his knowledge, solely by the test of one or more rigorous public examinations. This regulation would protect the examiners from all suspicion of interested motives, and from that kind of obloquy, to which the College of Surgeons have voluntarily subjected themselves, by requiring that candidates for diplomas shall have attended certain courses of lectures, delivered by members of their own body, or by individuals approved of by them.

Although we should consider it highly essential, that the acquirements of candidates should be ascertained by personal examinations, and not by tickets or certificates, we think it would be a proper preliminary, to any person's name being inscribed on the list of candidates for examination, that he should be required to produce testimonials of unexceptionable moral conduct and habits. These, it will be readily admitted, are, and ought to be, essential ingredients in the characters of medical men, but they do not admit, like professional qualifications, of being ascertained by a personal examination of the individual, and can only be truly certified, by persons who have had opportunities of observing the conduct of the parties for a length of time. Testimonials of this kind should, therefore, be required to proceed from the masters of such candidates, as might have served apprenticeships of several years, or from other persons whose known character and integrity, or whose public functions and official stations might be a guarantee of the fidelity of their certificates.

Were such a plan, as we have here sketched, to be acted upon, it would, of course, be proper to provide, that any person practising midwifery, without a license or diploma from the Board of Examiners, should be punishable, by fine, in the first instance, and by fine and imprisonment, if the practice should be persisted in. We are aware that the preference, for the attendance of female practitioners, which is still felt by some women, and which, perhaps, continues to prevail generally in some districts of the country, might be mentioned, as an objection, against any law such as is here suggested. This circumstance, however, is not entitled to so much weight, as might be supposed at the first mention of it. We have, for years past, had many opportunities of hearing women, who had been attended, in different labours, by practitioners of both sexes, express their decided preference for the assistance of male attendants, and their belief, that no person, who had been once attended by a gentleman, would ever think of having a midwife again. We are likewise informed, that in some districts of the country, especially in some parts of Scotland, where, till within the last twenty years, it was a rare occurrence, for any female of any rank, to employ a male accoucheur, the prejudice against this custom has so completely disappeared, that it is now very rare for midwives to be employed. Every gentleman, who has been much engaged in the practice of midwifery, must be aware, that, in many minute and even apparently trivial circumstances, a humane practitioner is enabled, by means of his professional knowledge, to alleviate the sufferings, and to contribute to the comfort of his patient; while, in the most essential particulars, he would be utterly incompetent to the due discharge of his duty, were it not for his acquaintance with anatomy, and with the changes to which the functions of the living system are liable, as illustrated in the doctrines of physiology and pathology. Let it be remembered, on the other hand, that the uninstructed women, who frequently practise midwifery, are often apt to flatter themselves, that they are discharging the functions of their office, in the most meritorious manner, by making frequent examinations, and practising rude and even painful manipulations of the parts concerned, at the same time exciting their patient to waste her strength, in useless exertions, when, perhaps, the circumstances of the case would require, that she should be left undisturbed, and that the practitioner should be waiting, in judicious inactivity, for the period to interfere with effect for her relief. All the practical knowledge that can belong to the generality of midwives, must consist in an empirical acquaintance with facts frequently presented to their observation, and impressed upon their memories in the course of practice, or handed by tradition from one lying-in room to another; and, although this kind of knowledge may possess a certain value, though defiled with many gross prejudices, yet, it must be admitted, that regularly educated medical practitioners are much better qualified to extract what is useful from what is hurtful, and to turn the whole to a good account, for the benefit of their patients, than the most respectable female can possibly be. For these substantial reasons we believe that the preference for the assistance of male practitioners must prevail, and increase until it shall probably become almost universal; yet we should be the last to propose any law to deprive females of the right of rendering their assistance as midwives, whenever the prejudices or predilections of individual patients, or their friends, demanded it. This could be productive of comparatively little mischief, if male practitioners were properly educated; because, in the rare instances, where females would be employed, the parties, being aware of their incompetency to manage cases of extraordinary difficulty or danger, would generally be prepared to have recourse, in any such cases, to proper medical assistance. The case is very different when a male practitioner is employed, as more implicit confidence is usually placed in him; and, though he may have neither bestowed any particular attention on the study of midwifery, nor undergone any examination as to his competency to practice it, and may have been impelled to engage in that practice merely from interested motives, yet, if possessed of a daring and unfeeling mind, as in the two cases quoted above, he may persevere to the discredit of the art of midwifery, and the disgrace of the profession of medicine, in pursuing it with impunity. We should therefore rejoice, (and in this feeling we have no doubt that a great majority of the profession will participate) if some such regulations as we have now ventured to suggest, were to be forthwith carried into execution.

*** Owing to the indisposition of the writer of this reviewal, and his consequent absence from town while it was passing the press, some typographical errors have unfortunately crept into that part of it contained in our last number, of which the reader is requested to correct the following:

Page 398, last line, *for* lines, *read* eines neuen.
399, line 13, *for* laud physicus, *read* land physicus.
404, first line, *for* naturally, *read* morally.
407, line 10 from the bottom, *for* tightening, *read* lightening.
407, line 11 from ditto, *for* sides, *read* edges.
427, last line, *for* five, *read* two.
430, line 11 from the bottom, *for* head, *read* bed.
434, line 21, *for* in, *read* on the machines in Dr. Davis's Theatre.
N.B. These words should have been included in the sentence preceding them.

